# Supplementary material for: Rice PIN Auxin Efflux Carriers Modulate the Nitrogen Response in a Changing Nitrogen Growth Environment
Source: Int J Mol Sci. 2021 Mar 23;22(6):3243. doi: 10.3390/ijms22063243 (PMC8005180; doi:10.3390/ijms22063243)
Supplement: Supplementary file 1 [file ijms-22-03243-s001.zip › Table S1. Information of the PIN family genes of rice, Arabidopsis and Maize..pdf]

Table S1. Information of the PIN family genes of rice, Arabidopsis and Maize.

Species	Group	Gene symbol	LOC_ID
Maize	group 1	ZmPIN1b	GRMZM2G074267
		ZmPIN1a	GRMZM2G098643
		ZmPIN1c	GRMZM2G149184
		ZmPIN1d	GRMZM2G171702
	group 2	ZmPIN2	AZM5_105354 & AZM5_3988
	group 4	ZmPIN8	GRMZM5G839411
	group 3	ZmPIN5b	GRMZM2G148648
		ZmPIN5c	GRMZM2G040911
		ZmPIN5a	GRMZM2G025742
	group 5	ZmPIN9	GRMZM5G859099
	group 6	ZmPIN10a	GRMZM2G126260
		ZmPIN10b	GRMZM2G160496
Rice	group 3	PIN5b	LOC_Os08g41720
		PIN5c	LOC_Os09g32770
		PIN5a	LOC_Os01g69070
	group 5	PIN9	LOC_Os01g58860
	group 4	PIN8	LOC_Os01g51780
	group 6	PIN10a	LOC_Os01g45550
		PIN10b	LOC_Os05g50140
	group 2	PIN2	LOC_Os06g44970
	group 1	PIN1b	LOC_Os02g50960
		PIN1a	LOC_Os06g12610
		PIN1c	LOC_Os11g04190
		PIN1d	LOC_Os12g04000
Arabidopsis	group 2	AtPIN2	AT5G57090
	group 1	AtPIN1	AT1G73590
	group 8	AtPIN6	AT1G77110
	group 4	AtPIN8	AT5G15100
	group 3	AtPIN5	AT5G16530
	group 7	AtPIN7	AT2G01420
		AtPIN3	AT1G70940
		AtPIN4	AT1G23080
