# Supplementary material for: Rice PIN Auxin Efflux Carriers Modulate the Nitrogen Response in a Changing Nitrogen Growth Environment
Source: Int J Mol Sci. 2021 Mar 23;22(6):3243. doi: 10.3390/ijms22063243 (PMC8005180; doi:10.3390/ijms22063243)
Supplement: Supplementary file 1 [file ijms-22-03243-s001.zip › Table S2. Summary of primer sequences used for RT-PCR analyses in Figures 1, 3, and 6..pdf]

Table S2. Summary of primer sequences used for RT-PCR analyses in Figures 1, 3, and 6.

Purpose	Gene symbol	LOC_ID	Forward primer(5'-3')	Reverse primer(5'-3')
Genotyping	OsPIN1b	LOC_Os02g50960	CAC GGT GAC GTG TAT CCT GC	GCC GGA AGC AAT GTC TCT CA
RT-PCR	PIN5b	LOC_Os08g41720	GCA AAG GAG TAT GGG CTT CA	TCT AAC ATG CAT TTA CAG CA
RT-PCR	PIN5c	LOC_Os09g32770	CTG CTC GTG ATG AAC AGG CT	ATA CTC AGC ACA GCG GTT AT
RT-PCR	PIN5a	LOC_Os01g69070	TAG AGC TAA TTA GGT AGA CG	ACT GTA ATG GGA GTA TCG AG
RT-PCR	PIN9	LOC_Os01g58860	TGG TCA TCA AGT TTC TGA TA	ACA TCA TGA GCA CAG GGG TT
RT-PCR	PIN8	LOC_Os01g51780	TGC TGA AGA TAG CCA TTG TA	TAT TCT GAG CAC TGC GAT AA
RT-PCR	PIN10a	LOC_Os01g45550	TTA TGG ACG CTC TCA CAT AA	TAT CTC TGC GAC ATT TGT AC
RT-PCR	PIN10b	LOC_Os05g50140	AGG CAC GCT CTT GCG TAT CG	CCA TCA CCT TGG TCT ACT AT
RT-PCR	PIN2	LOC_Os06g44970	GTT CAT GGC TCT GCA ACC AA	TTG TCC CGT TTG TGT TTG C
RT-PCR	PIN1b	LOC_Os02g50960	TGC ACC CTA GCA TTC TCA GCA	GAA GTA GAA TTT GGG AGG AGG G
RT-PCR	PIN1a	LOC_Os06g12610	ATC TGG TCG CTC GTC TGC TT	AAG GTG GCG ACG TTC GCC AT
RT-PCR	PIN1c	LOC_Os11g04190	TGC ACA TTG CCA TTG TTC AG	TGA TAT TCG GGA TGC TGA TT
RT-PCR	PIN1d	LOC_Os12g04000	GTG CTT CTG CAC ATT GCC AT	TGA TAT TCG GGA TGC TGA TA
RT-PCR	GS1;2	LOC_Os03g12290	ACA TGG ACC CAT ACG TCG TC	TGA TGT GTC AAC GTT GGA TT
RT-PCR	AMT1;2	LOC_Os02g40730	GGA GGC CAT GTC TAG TGG AA	TGC AGG TTT CGT GAT TGT AT
RT-PCR	AMT1;1	LOC_Os04g43070	TTC GCG TAC GTC TAC CAC GA	GGC TGC CTC CAA CAG CAA CA
RT-PCR	NADH-GOGAT1	LOC_Os01g48960	TGC AGC TGT CGA CAA GTA CT	TGA TCA CAG CAA GTT GTC TT
RT-PCR	AMT1;3	LOC_Os02g40710	GCA CAT CGT GCA GAT CCT GG	ATG GAA GGC ATG GAC CCG AC
RT-PCR	GLU3	LOC_Os04g41970	TAC ATG GAG TCC GTC AAC GT	GTA CGG CAA GAA GTA CCC GC
RT-PCR	GDH2	LOC_Os04g45970	ATT ACC TGA CAT TTA CGC CA	GAT TGC AAC CTG AGG ATG GG

RT-PCR	AS2	LOC_Os06g15420	CGC TAG CTT TGT TTA CCC AG	TTG AAT GGG ACG CAG CCT GG
