# Supplementary figures and images for: Rice PIN Auxin Efflux Carriers Modulate the Nitrogen Response in a Changing Nitrogen Growth Environment
Source: Int J Mol Sci. 2021 Mar 23;22(6):3243. doi: 10.3390/ijms22063243 (PMC8005180; doi:10.3390/ijms22063243)

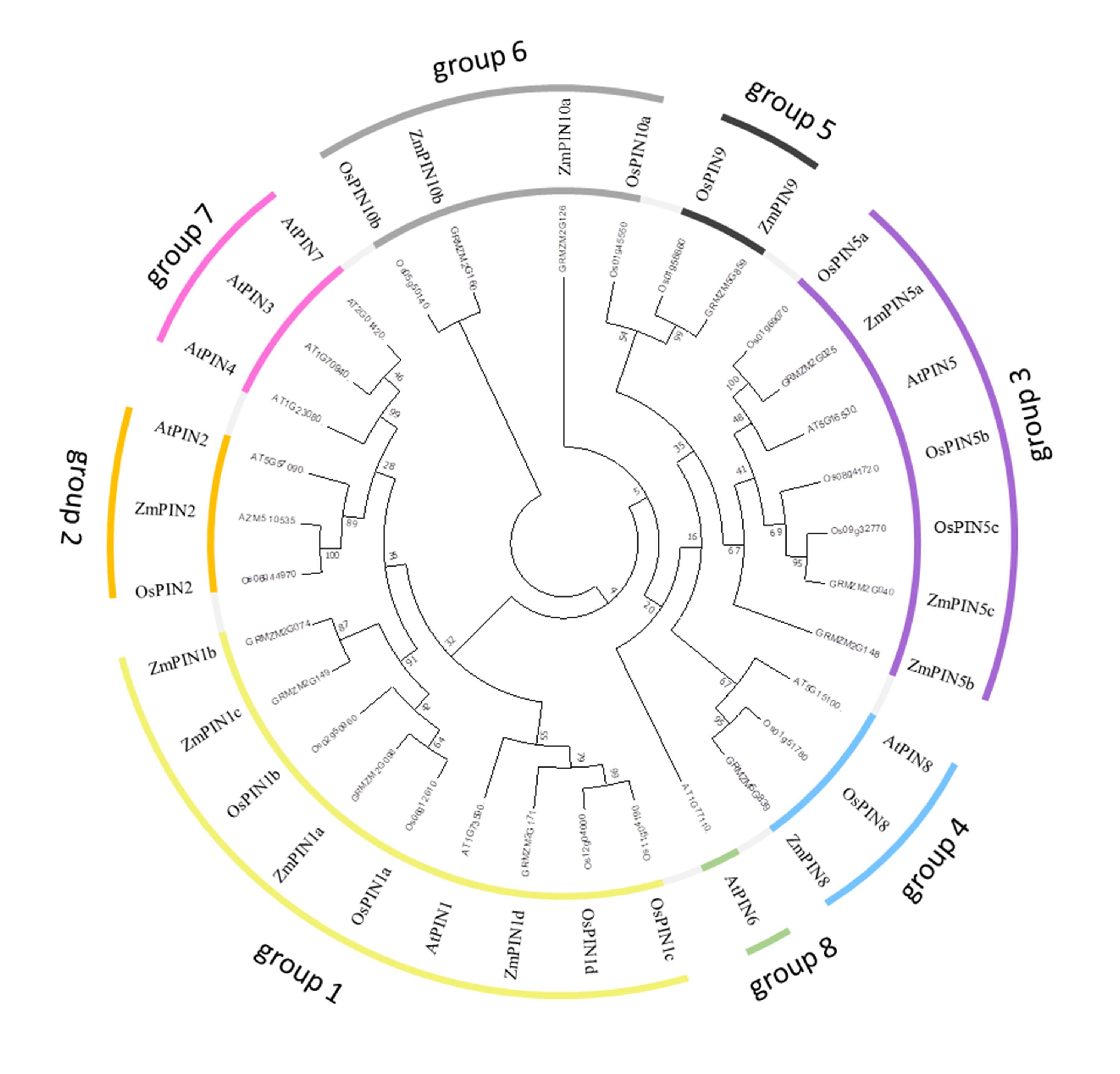

Supplement: Supplementary file 1 [file ijms-22-03243-s001.zip › Figure S1.tif]

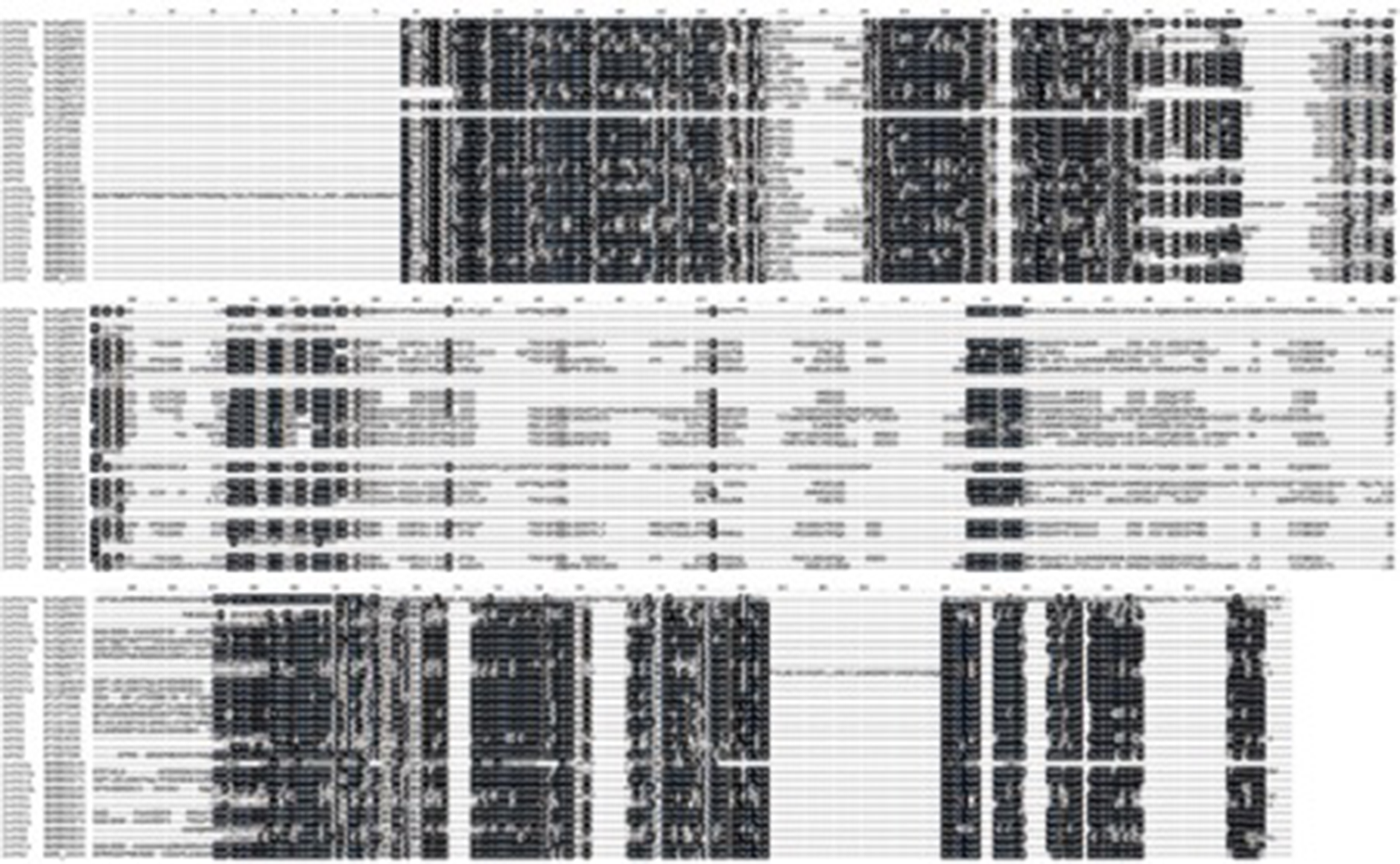

Supplement: Supplementary file 1 [file ijms-22-03243-s001.zip › Figure S2 protein align.tif]
